# Lipid Signatures Associated with Diabetic Peripheral Neuropathy in Obesity and Type 2 Diabetes—A Systematic Review

**DOI:** 10.3390/jcm15103976

**Published:** 2026-05-21

**Authors:** Cristina Mocanu (Chitan), Teodor Salmen, Marius-Costin Chitu, Radu-Cristian Cimpeanu, Simona Clus, Delia Reurean-Pintilei, Anca Pantea Stoian, Cristian Serafinceanu

**Affiliations:** 1 Doctoral School, Carol Davila University of Medicine and Pharmacy, 020021 Bucharest, Romania; 2 Outpatient Diabetes Center 1, Suceava County Emergency Clinical Hospital “Sf. Ioan cel Nou”, 720224 Suceava, Romania; 3 Department of Emergency Medicine, Clinical Emergency County Hospital, 200642 Craiova, Romania; 4 Faculty of Medicine and Biological Sciences, “Ştefan cel Mare“ University, 720229 Suceava, Romania; simona.clus@usm.ro; 5 Department of Medical-Surgical and Complementary Sciences, Faculty of Medicine and Biological Sciences, “Ștefan cel Mare” University, 720229 Suceava, Romania; delia.pintilei@usm.ro; 6 Department of Diabetes, Nutrition and Metabolic Diseases, Consultmed Medical Center, 700544 Iasi, Romania; 7 Department of Diabetes, Nutrition and Metabolic Diseases, “Carol Davila” University of Medicine and Pharmacy, 020021 Bucharest, Romania; anca.stoian@umfcd.ro (A.P.S.); cristian.serafinceanu@umfcd.ro (C.S.)

**Keywords:** diabetic peripheral neuropathy, type 2 diabetes mellitus, obesity, lipidomics, dyslipidemia, hypertriglyceridemia, acylcarnitine, sphingolipids, mitochondrial dysfunction, microvascular impairment, systematic review

## Abstract

**Background and Objectives:** Diabetic peripheral neuropathy (DPN) is a common and debilitating complication of obesity and type 2 diabetes (T2D) affecting up to 50% of patients with long-standing disease. While chronic hyperglycemia plays a central role in its pathogenesis, intensive glycemic control provides only partial protection, suggesting the involvement of additional metabolic pathways. The primary objective of this systematic review was to evaluate the role of lipid metabolism disturbances and advanced lipidomic alterations in the development and progression of DPN in patients with obesity and T2D. Secondary objectives included identifying specific lipid species associated with DPN and exploring their potential pathophysiological and clinical implications. **Methods:** This systematic review included 8 studies that met the inclusion criteria and was conducted according to PRISMA guidelines and registered in PROSPERO/2026/CRD420261288920. Study quality was assessed using the Newcastle–Ottawa Scale. **Results:** Large population-based cohorts reported a consistent association between hypertriglyceridemia and DPN prevalence, with triglyceride levels >204 mg/dL associated with an approximately 40% increased risk. Lipidomic analysis revealed alterations in acylcarnitine, sphingolipids, and phospholipids. However, the evidence remains limited and heterogeneous, and neuropathy-specific outcomes were insufficiently evaluated in interventional studies. **Conclusions:** Lipid metabolism disturbances, particularly hypertriglyceridemia and specific lipidomic alterations, may contribute to DPN beyond the effects of hyperglycemia. Although not yet clinically actionable, lipidomic alterations may represent promising future biomarkers and therapeutic targets in DPN. However, the current evidence is limited by heterogeneity and predominantly observational designs. Further well-designed longitudinal and interventional studies are needed to clarify causal relationships and clinical relevance.

## 1. Introduction

Type 2 diabetes (T2D) represents a major global public health challenge, with a continuously increasing prevalence worldwide [1,2]. According to the International Diabetes Federation, 588.7 million adults were living with diabetes mellitus (DM) in 2024, a number projected to reach 852.5 million by 2050 [1]. This growing burden is largely driven by aging populations, urbanization, and lifestyle-related factors, placing significant pressure on healthcare systems [1,2].

In Romania, T2D, according to the national PREDATORR study and the International Diabetes Federation, shows a positive trend, with many undiagnosed cases, underscoring its considerable impact and the need for improved screening, prevention, and management strategies [3].

Diabetic peripheral neuropathy (DPN) is one of the most common chronic complications of DM, affecting up to 50% of individuals with long-standing disease [4,5]. However, reported prevalence estimates vary considerably across studies due to heterogeneity in diagnostic criteria and assessment methods, including differences in symptom-based evaluations, neurological examination, nerve conduction studies, and small-fiber neuropathy testing. Characterized by progressive sensory loss, neuropathic pain, and autonomic dysfunction, it represents a major cause of foot ulceration, lower-limb amputation, impaired quality of life, and increased cardiovascular (CV) morbidity and mortality [5,6,7]. Despite advances in DM management, the global burden of DPN continues to increase, paralleling the rising prevalence of obesity and CV disease worldwide [4].

Although hyperglycemia remains the central mechanism implicated in DPN pathogenesis, increasing evidence suggests that lipid dysregulation may also contribute substantially through pathways involving mitochondrial dysfunction, oxidative stress, neuroinflammation, and impaired neuronal membrane integrity [5,6,7,8,9,10,11].

Obesity and T2D are frequently accompanied by profound disturbances in lipid metabolism, including elevated triglycerides (TG), increased circulating free fatty acids (FFA), altered lipoprotein profiles, and qualitative changes in lipid composition [9]. Beyond their established role in atherosclerotic CV disease, lipids have been implicated in mitochondrial dysfunction, oxidative stress, inflammation, and microvascular impairment—processes that are also central to peripheral nerve damage [12,13,14,15,16]. Observational studies have reported independent associations between adverse lipid profiles and DPN prevalence or severity, even after adjustment for glycemic indices [11,12,13].

In parallel, emerging lipidomic studies have identified alterations in acylcarnitines, sphingolipids, and phospholipids associated with neuropathy in patients with T2D and obesity [13,14,15,16,17,18]. Evidence suggests that these signatures may precede the clinical manifestation of DPN and correlate with objective measures of nerve fiber loss, neuropathic pain, and autonomic dysfunction [14,18]. Nevertheless, the available data remain heterogeneous, with substantial variability in study design, patient populations, lipidomic methodologies, and neuropathy assessment tools.

Importantly, DPN shares common pathophysiological pathways with CV disease, including endothelial dysfunction, arterial stiffness, and chronic low-grade inflammation. As a result, lipid abnormalities associated with DPN may also serve as markers of broader CV risk [15]. Despite this overlap, lipidomic characteristics linked to DPN have not been systematically synthesized, and their potential role in risk stratification and disease modification remains insufficiently defined [16].

Although dyslipidemia has been increasingly implicated in DPN pathogenesis, existing evidence remains fragmented and heterogeneous, particularly in integrating conventional lipid abnormalities with emerging lipidomic alterations. Moreover, the relationship between lipidomic signatures and clinically relevant neuropathy outcomes in patients with T2D and obesity has not been systematically synthesized. Therefore, this systematic review aimed to evaluate current evidence regarding the association between conventional lipid disturbances and advanced lipidomic alterations with DPN in patients with T2D and obesity. In addition, this review sought to explore potential mechanistic pathways, clinical implications, and future directions for biomarker research in this field.

## 2. Materials and Methods

This systematic review was conducted in accordance with the Preferred Reporting Items for Systematic Reviews and Meta-Analyses (PRISMA) guidelines (Supplementary Table S1) [19]. The protocol for this review has been registered with the identifier PROSPERO/2026/CRD420261288920.

### 2.1. Research Question and Search Strategy

The research question was framed using the Population, Exposure, Comparison, and Outcome (PECO) framework. The population included adult patients with T2D and obesity. The exposure of interest was lipid metabolism disturbances, including both conventional lipid parameters and lipidomic alterations. A comparison was made between patients with and without DPN at different levels of disease severity. The outcome included the presence, severity, or progression of DPN.

A comprehensive search strategy was conducted in PubMed, Scopus, and Web of Science databases for studies published between 1 January 2014 and 15 April 2026. The search strategy combined Medical Subject Headings (MeSH) and free-text terms including (“diabetic polyneuropathy” OR “DPN”) AND (“dyslipidaemia” OR “lipid metabolism imbalance” OR “lipid metabolism disturbance”) for each of the three databases, with each database-specific search string and filters used, as follows:

- “(diabetic polyneuropathy OR DPN) AND (dyslipidaemia OR lipid metabolism imbalance OR lipid metabolism disturbance)” search string with “2016–2026”, “Article”, and “English” filters for the Scopus database.
- “dyslipidaemia OR lipid metabolism imbalance OR lipid metabolism disturbance” search string with “in the last 10 years”, “Article”, and “English” filters for the PubMed database.
- “dyslipidaemia OR lipid metabolism imbalance OR lipid metabolism disturbance” search string with “2016–2026”, “Article”, and “English” filters for the Web of Science database.

### 2.2. Inclusion Criteria

Studies were included if they met the following criteria:

(1) Published in English.
(2) Original full-text articles (cohort or cross-sectional studies).
(3) Conducted on adult human populations.
(4) Published in the last ten years.
(5) Included patients with obesity and T2D diagnosed with DPN. Obesity was defined according to World Health Organization criteria as a body mass index ≥30 kg/m^2^ [1,2]. T2D was defined based on established diagnostic criteria, including fasting plasma glucose ≥126 mg/dL, HbA1c ≥6.5%, or the use of glucose-lowering medication [7].
(6) Reported data on lipid parameters or lipidomic profiles in relation to DPN outcomes.

### 2.3. Exclusion Criteria

Studies were excluded if they:

(1) Were reviews, case reports, conference or meeting abstracts, editorials, letters to the editor, or expert opinions;
(2) Included duplicate or overlapping populations;
(3) Reported incomplete or unclear data;
(4) Focused exclusively on type 1 DM or studies not evaluating DPN or neuropathy-related outcomes;
(5) Were conducted in animal models or in vitro settings.

### 2.4. Data Extraction

Two reviewers independently extracted data using a standardized, pilot-tested data extraction form. The extraction information included study characteristics, population details, lipid parameters, lipidomic markers, and reported outcomes. Any differences of opinion were settled through discussion or consultation with a third reviewer. Independent screening demonstrated substantial agreement between reviewers (Cohen’s κ = 0.65), supporting the reliability of the study selection process.

### 2.5. Risk of Bias Assessment

The methodological quality of the included studies was assessed independently by two reviewers using the Newcastle–Ottawa Scale (NOS) [20]. Studies with a score of 6 or more were considered of moderate to high quality.

### 2.6. Strategy for Data Synthesis

The relatively small number of included articles (n = 8) reflects the limited body of literature specifically addressing lipid metabolism and lipidomic alterations in DPN within the combined context of obesity and T2D over the last decade. Also, strict inclusion criteria focusing on adult human populations with clearly defined DPN outcomes and lipid-related parameters contributed to the reduced number. Due to substantial heterogeneity across studies in terms of clinical heterogeneity (T2D duration, obesity status, DPN diagnostic criteria), methodology (study design—cross-sectional, cohort, interventional), lipid parameters evaluated, statistical adjustments, and outcome assessment approaches of DPN (ranging from clinical scoring systems to nerve conduction studies and intraepidermal nerve fiber density measurements), a quantitative meta-analysis was not considered appropriate. Instead, a narrative synthesis was performed, focusing on the qualitative integration of findings across studies.

Due to this heterogeneity and the limited number of studies, quantitative synthesis and formal I^2^ estimation were not considered methodologically appropriate.

Publication bias could not be formally assessed using funnel plots because quantitative synthesis was not feasible due to substantial methodological heterogeneity and the limited number of eligible studies.

The search strategy was designed to prioritize specificity over sensitivity to identify studies directly evaluating the relationship between DPN and lipid metabolism disturbances. Because the review focused specifically on clinically relevant DPN in the context of T2D and obesity, narrower terminology was intentionally applied to reduce the retrieval of unrelated neuropathy and metabolic studies. The search was limited to studies published within the last 10 years to capture contemporary evidence reflecting recent advances in lipidomic profiling technologies and current approaches to DPN assessment and metabolic characterization. The inclusion criteria were intentionally designed to prioritize studies directly evaluating DPN in the context of lipid metabolism disturbances and lipidomic alterations in patients with T2D and obesity. However, this stringent approach may have reduced the number of eligible studies and contributed to the limited evidence base. The relatively low duplication rate identified during the screening process was likely attributable to the highly specific search strategy and narrowly defined eligibility criteria applied across databases. In addition, differences in database indexing and keyword combinations may have further reduced record overlap. Duplicate identification and removal were independently verified using a structured deduplication workflow to ensure methodological rigor and transparency.

## 3. Results

### 3.1. Study Selection

**Figure 1:**
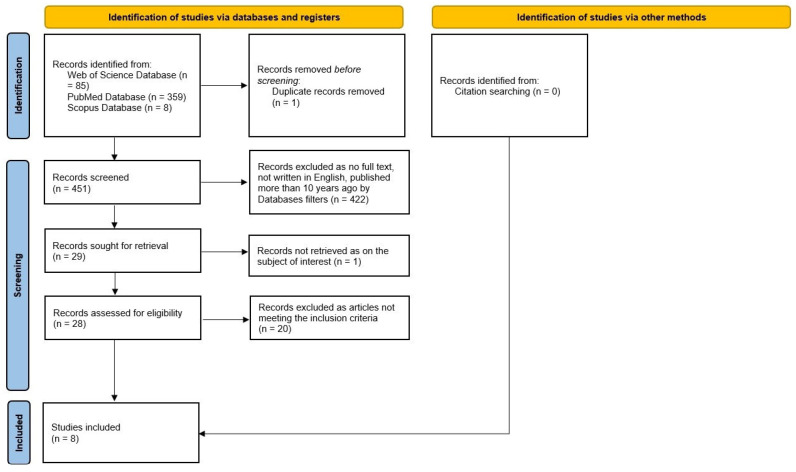
Flowchart of the study selection process.

The literature search from 15 April 2026 identified 452 records (359 from PubMed, 8 from Scopus, and 85 from Web of Science) that underwent the study selection process, as summarized in Figure 1 (PRISMA 2020 flow diagram), which also details the identification, screening, eligibility, and inclusion stages.

After removing 1 duplicate, 451 records were screened in each of the three databases using filters for language (English), publication type (original articles), and date range (2014 to the date of the search). When the 29 records were sought for retrieval, 1 more record was eliminated as not being on the subject of interest.

Following the screening process, 28 reports were assessed for eligibility by two independent reviewers, with a third reviewer resolving any disagreements. Eight studies published between 2014 and 2026 were included for qualitative synthesis.

Research papers rated at least 6 stars on the NOS scale are considered of moderate to high quality and covered in Section 3, and the selection process is summarized in Table 1.

### 3.2. Lipid Parameters and DPN in Obesity

In a cohort study by Callaghan et al. [25], TG levels showed a weak association with neuropathy outcomes, with confidence intervals crossing unity in several analyses. Similarly, HDL-C demonstrated a potential inverse association with intraepidermal nerve fiber density, although the strength of this relationship was limited.

In a larger cohort study [24] evaluating components of metabolic syndrome, elevated TG levels were associated with prevalent neuropathy, whereas associations with incident neuropathy were not statistically significant. HDL-C showed a non-significant inverse relationship.

An interventional study [26] assessing the effects of free fatty acid (FFA) reduction reported a decrease in plasma FFA levels following treatment; however, the change was not statistically significant in the obesity subgroup.

The characteristics and parameters of interest for the studies with adult patients with obesity and DPN are synthesized in Table 2.

### 3.3. Lipid Parameters and DPN in T2D

A large cohort study [20] demonstrated that TG levels > 204 mg/dL were associated with an increased prevalence of DPN. Similarly, a population-based analysis [21] reported that multiple metabolic factors contributed to DPN burden, with TG, LDL-C, and body mass index showing smaller, measurable contributions compared to glycemic control and blood pressure.

The characteristics and parameters of interest for studies of adult patients with T2D and DPN are summarized in Table 3.

### 3.4. Lipidomic Alterations

A study [16] investigating acylcarnitine profiles reported significant reductions in long-chain acylcarnitine, including ximenoylcarnitine (26:1) and lignoceroylcarnitine (24:0), in patients with T2D and DPN (*p* < 0.05). In addition, changes in sphingolipid species, including hydroxylated sphingolipids, were observed.

Another Mendelian randomized study [21] that evaluated 179 lipid species identified specific phosphatidylcholine species as associated with DPN risk. Higher genetically predicted levels of the species 16:0_20:2 and 16:1_18:1 were associated with a reduced risk of DPN.

These findings indicate alterations in lipid metabolism pathways, particularly those related to mitochondrial function and cellular membrane composition.

### 3.5. Effect of Free Fatty Acid Modulation

An interventional study [27] evaluating the effects of acipimox on 22 participants demonstrated a significant reduction in fasting plasma FFA levels in patients with T2D, but only after 2 weeks of follow-up, so the results should be interpreted cautiously.

However, neuropathy-specific outcomes were not consistently assessed, limiting the interpretation of the clinical relevance of these findings.

## 4. Discussion

This systematic review provides a focused synthesis of the relationship between conventional lipid abnormalities, advanced lipidomic alterations, and DPN in the specific context of obesity and T2D. Unlike previous narrative discussions primarily centered on dyslipidemia or glycemic injury alone, this review integrates epidemiological evidence with emerging mechanistic lipidomic data, including alterations in TG, FFA, acylcarnitines, sphingolipids, and phospholipids. To our knowledge, this is the first systematic review specifically integrating conventional lipid parameters with advanced lipidomic alterations in DPN within the combined context of obesity and T2D.

In addition, this review highlights the convergence of metabolic, mitochondrial, inflammatory, and neurovascular pathways potentially linking lipid dysregulation to peripheral nerve injury beyond hyperglycemia alone. Finally, the review identifies major methodological gaps in the current literature, including heterogeneous neuropathy definitions, variable lipidomic methodologies, and the predominance of observational designs, thereby outlining priorities for future translational and longitudinal research. The proposed framework linking lipid dysregulation and lipidomic alterations with potential mechanisms involved in DPN is summarized in Table 4.

### 4.1. Interpretation of Heterogeneity

The heterogeneity across included studies reflects differences in study design (cross-sectional, cohort, and interventional studies); sample size (22 included patients to nearly 1 million patients); population characteristics (obesity status, T2D duration, glycemic control, and cardiometabolic comorbidity burden); DPN assessment (clinical diagnosis, nerve conduction studies, and intraepidermal nerve fiber density); lipid parameters evaluated (traditional lipidic profile versus advanced lipidomics); and statistical adjustments rather than purely contradictory biological findings.

Overall, large population-based studies consistently associate dyslipidemia, specifically hypertriglyceridemia, as a relevant risk factor beyond glycemic control. At the same time, smaller mechanistic lipidomic investigations provide biological plausibility by demonstrating alterations in acylcarnitines, sphingolipids, and phospholipids, leading to disrupted mitochondrial β-oxidation and neuronal membrane homeostasis.

The strength and consistency of this association vary substantially across studies, and the evidence remains limited by heterogeneity in study design, population characteristics, and outcome assessment methods. Emphasis should be placed on the need for cautious interpretation of the results and on the importance of adequately powered longitudinal studies that integrate population-level epidemiology with molecular lipidomic characterization. Finally, this study integrates lipidomics with clinical DPN, generating hypotheses such as the supposition that lipid dysregulation may contribute to DPN through interconnected metabolic, inflammatory, and neurovascular pathways and provide insights for future biomarker research.

### 4.2. Association Between Traditional Lipid Parameters and Neuropathy

Several studies evaluated the relationship between conventional lipid markers—TG, HDL-C, LDL-C, and total cholesterol—and outcomes of neuropathy. In obesity-focused cohorts, Callaghan et al. (2016) observed modest associations between TG levels and neuropathy; however, they had limited statistical robustness [25,26]. Interestingly, HDL-C showed a negative association with intraepidermal nerve fibers, suggesting a potential structural impact of lipid imbalance on small fiber integrity. In a larger metabolic syndrome cohort, elevated TG levels were associated with neuropathy without significant prediction of incident neuropathy, suggesting that dyslipidemia may be strongly associated with prevalent rather than incident neuropathy. Diabetic microangiopathy is a critical component of DPN pathophysiology [5,9,27,28,29,30,31,32,33], leading to the so-called “capillary steal” phenomenon [5,9,27,28,29,30,31,32,33], thereby contributing to nerve ischemia and delayed wound healing [5,29,34,35,36]. Even if they do not directly initiate diabetic foot ulcers, they significantly influence ulcer progression and repair capacity [1,30,37,38]. Neuromodulatory approaches and systemic treatments, such as hyperbaric oxygen therapy, represent lipid-modulating therapies that aim to improve tissue oxygenation, attenuate oxidative stress, and enhance wound healing in diabetic foot syndrome [5,30,31,39,40,41]. Whether conventional lipid-lowering agents are sufficient to reverse established neuropathy remains uncertain, particularly given evidence of mitochondrial and lipidomic remodeling beyond standard lipid parameters [31,37,41,42,43].

In contrast, Afshinnia et al. (2022) did not observe significant differences in total cholesterol or TG levels between T2D patients with and without neuropathy [24]. This discrepancy may reflect limited sample size (n = 69) and reduced statistical power, underscoring the importance of adequately powered cohort studies [23].

Large population-based studies provided stronger epidemiological support. Kristensen et al. (2024), analyzing over 60,000 individuals with T2D, demonstrated that TG levels > 204 mg/dL were associated with a 40% increase in DPN prevalence after adjustment [22]. Similarly, Lin et al. (2023) reported that LDL-C ≥ 1.8 mmol/L was associated with neuropathy burden, although hyperglycemia and HBP had higher population attributable fractions [23]. These large datasets reinforce the relevance of dyslipidemia as a modifiable risk factor, albeit secondary to glycemic control [44,45].

Collectively, the evidence suggests that hypertriglyceridemia and possibly low HDL-C are associated with neuropathy risk, particularly in large, population-based studies. However, causality remains uncertain, and lipid abnormalities are likely to act synergistically with hyperglycemia, insulin resistance, and inflammatory mechanisms in the development and progression of DPN [3].

### 4.3. Lipidomic Alterations—Mechanistic Insights

Emerging lipidomic evidence suggests that DPN is associated with profound alterations in lipid species involved in mitochondrial function, membrane integrity, and cellular signaling. Specifically, reductions in long-chain acylcarnitines and alterations in sphingolipid composition indicate impaired mitochondrial β-oxidation, reduced ATP production, and increased oxidative stress. These mechanisms are biologically plausible and align with known pathways of neuronal injury, including axonal degeneration and neuroinflammation. Beyond traditional lipid panels, advanced lipidomic analyses revealed qualitative alterations in lipid species associated with DPN.

Rumora et al. (2021) identified significant reductions in long-chain acylcarnitine (e.g., ximenoylcarnitine and lignoceroylcarnitine) and hydroxylated sphingolipids in patients with T2D and DPN [16]. These findings suggest disruptions in mitochondrial fatty acid β-oxidation and sphingolipid metabolism, both of which are critical for neuronal membrane integrity and energy homeostasis. Given the multifactorial and heterogeneous nature of DPN pathogenesis—including metabolic dysregulation, vascular impairment, neuroinflammation, neurotrophic deficits, impaired nerve regeneration, and potential genetic susceptibility—a comprehensive understanding of lipidomic perturbations and their mechanistic contribution to neuropathic pain is essential [9,31,32,33,42,45].

Several included studies identified alterations in ceramide and sphingolipid metabolism in patients with DPN, suggesting a potential role for these lipid species in metabolic and neuronal dysfunction. Additional mechanistic evidence from external experimental and epidemiological literature has further implicated ceramide and sphingolipid dysregulation in pathways related to oxidative stress, mitochondrial dysfunction, inflammation, and peripheral nerve injury. However, the current systematic synthesis does not provide sufficient evidence to establish quantitative risk estimates or causal relationships between specific lipid species and DPN development [14,23].

From the lipid species that are linked to DPN in T2D patients, not only phosphatidylcholine increased the risk [21], but other species also proved to be significantly different between groups—sphingosine (d18:0), carnitine 22:1, lysophosphatidylethanolamine (LPE) (18:0/0:0), lysophosphatidylcholine (LPC) (16:0/0:0), LPC (18:1/0:0), LPC (0:0/18:0), and LPE (0:0/18:1)—and were even correlated with electromyography results: sphingosine (d18:0), carnitine 22:1, LPE (18:0/0:0), and LPC (0:0/18:0) [46].

Longitudinal cohort data indicate that specific lipidomic signatures, including altered acylcarnitines and sphingolipids, may precede the development of clinical neuropathy by several years, suggesting potential utility as early biomarkers for DPN risk stratification [15,33].

### 4.4. Lipidomic Alterations: Clinical Utility

Despite these mechanistic insights, the clinical applicability of lipidomic profiling remains limited. Currently, lipidomic analyses lack standardization, are not widely accessible, and have not been validated in large, diverse populations. Therefore, their role in routine clinical practice, including risk stratification and therapeutic targeting, remains uncertain and is primarily confined to research settings.

Also, these findings support the hypothesis that neuropathy is not solely associated with elevated circulating lipid concentrations but may involve deeper metabolic remodeling at the cellular level [4,17,32,46,47,48], and they also provide genetic support for potential hypothesis generation regarding specific lipid species associated with DPN. However, the directionality and biological mechanisms require further investigation [21].

Importantly, several lipidomic alterations identified across the included studies demonstrated potential clinical relevance. Altered TG metabolism, elevated FFA levels, and sphingolipid abnormalities were associated with markers of neuropathic severity, impaired glycemic control, reduced nerve fiber integrity, and increased cardiometabolic risk. These findings suggest that lipidomic profiling may not only contribute to mechanistic understanding but may also support future biomarker development for disease progression, therapeutic monitoring, and risk stratification in patients with obesity and T2D.

### 4.5. Effects of Free Fatty Acid Modulation

One interventional study by Daniele et al. (2014) showed that targeting lipid metabolism pharmacologically may influence metabolic pathways implicated in neuropathy, as assessed by acipimox [27]. This nicotinic acid derivative reduces plasma FFA in T2D patients. Given a follow-up of only 2 weeks and significantly lower fasting plasma FFA levels, this study suggests only that modulation of lipid flux is feasible and should therefore be interpreted with caution due to limited statistical power, a short follow-up, and the lack of neuropathy-specific outcomes [26].

The modulation of FFA metabolism represents an important mechanistic component linking obesity, insulin resistance, and DPN. Although the interventional findings included in this review demonstrated only modest reductions in FFA levels, these observations remain biologically relevant because chronic lipid overload may promote sustained metabolic and inflammatory injury within peripheral nerves. Integrating FFA dysregulation into the broader lipidomic framework highlights its potential role as both a pathogenic mediator and a therapeutic target in obesity-associated T2D complications.

### 4.6. Obesity, Metabolic Syndrome, and Neuropathy

Obesity represents a major independent risk factor for peripheral neuropathy, even in the absence of overt T2D. Epidemiological evidence from recent cohort studies (2019–2026) consistently demonstrates that increased body mass index and central adiposity are significantly associated with subclinical and clinical peripheral nerve impairment, including reduced intraepidermal nerve fiber density and impaired sensory function. In multivariable-adjusted analyses, obesity and overweight have been associated with increased odds of neuropathy (adjusted odds ratio > 1), with statistically significant associations reported across multiple populations (*p* < 0.05), independent of glycemic status [49].

Within the metabolic syndrome framework, neuropathy risk appears to increase in a dose-dependent manner as metabolic abnormalities accumulate. Recent large-scale observational studies have shown a significant positive trend between the number of metabolic syndrome components and the likelihood of developing peripheral neuropathy (*p* for trend < 0.01). This supports a cumulative pathophysiological effect, in which dyslipidemia, insulin resistance, HBP, and central obesity interact synergistically to promote peripheral nerve injury [50].

Among individual metabolic components, dyslipidemia—particularly elevated TG and LDL-C—has been independently associated with neuropathic outcomes after adjustment for glycemic control, age, and T2D duration. Reported adjusted odds ratios for neuropathy in individuals with dyslipidemia remain consistently above unity across studies, with 95% confidence intervals excluding the null value in several multivariate models, indicating statistical significance (*p* < 0.05). These findings suggest that lipid abnormalities may contribute independently to neuropathic risk beyond hyperglycemia alone [50,51].

Mechanistically, the metabolic syndrome promotes neuropathy through interconnected pathways involving chronic low-grade inflammation, endothelial dysfunction, oxidative stress, and impaired mitochondrial bioenergetics. These processes result in progressive axonal injury and small fiber degeneration. Importantly, recent evidence supports the concept that metabolic dysfunction can induce neuropathic changes even before the onset of overt T2D, reinforcing the notion of “metabolic neuropathy” as a continuum rather than a diabetes-restricted complication [49,52].

Overall, contemporary literature supports a multifactorial model of neuropathy in obesity and metabolic syndrome, in which metabolic dysregulation confers a statistically significant increase in risk (OR > 1, *p* < 0.05) through additive and synergistic effects on vascular, inflammatory, and neuronal pathways [53].

The metabolic syndrome framework suggests a multifactorial pathogenesis in which dyslipidemia interacts with hyperglycemia, HBP, and systemic inflammation to promote nerve injury [54].

### 4.7. Clinical and Research Implications

From a clinical perspective, the association between TG levels and DPN, particularly in large population-based studies, suggests that lipid parameters may contribute to a broader cardiometabolic risk profile in patients with T2D and obesity. However, current evidence does not support the use of lipid markers as independent diagnostic or prognostic tools for DPN, and their role in routine clinical decision-making remains unclear. In this context, conventional lipid parameters, especially TG, may be considered part of an integrated risk assessment framework rather than specific markers of neuropathy, with clinical relevance interpreted within the overall metabolic profile, including glycemic control, blood pressure, and other established risk factors.

Advanced lipidomic profiling provides additional insights into potential mechanisms underlying DPN, particularly mitochondrial dysfunction and altered cellular signaling, that are currently limited to research settings due to a lack of standardization, high costs, and insufficient validation in large, diverse populations. As such, their immediate clinical applicability remains limited. Genetic evidence suggests that specific lipid species may represent potential targets for further mechanistic and interventional studies.

From a research perspective, the present findings highlight the need for more robust, methodologically consistent studies with longitudinal designs and standardized definitions and assessment methods for DPN, along with interventional studies targeting lipid metabolism to determine whether modifying lipid profiles can influence the development or progression of DPN. At present, the available evidence is insufficient to support specific lipid-modulation strategies for neuropathy outcomes, and the integration of molecular and clinical data is crucial. Bridging the gap between large epidemiological studies and small mechanistic lipidomic analyses represents a key challenge for the field.

Nonetheless, DPN management should be multidisciplinary and integrate not only metabolic control but also medical, lifestyle, and patient-centered care strategies. Structured T2D self-management education and support programs have been shown to improve glycemic control, adherence, and long-term outcomes [54]. In addition, community-based and nurse-led interventions contribute to significant reductions in HbA1c and improved risk control, highlighting the importance of coordinated care delivery models [55]. Psychosocial factors, including DM distress, health literacy, and patient-provider communication, further influence self-care behaviors and disease outcomes, underscoring the need for integrated clinical and educational strategies. In this context, lipid abnormalities should be addressed in multidisciplinary care approaches involving physicians, DM nurse specialists, and allied health professionals [55,56]. Such integrated models may facilitate earlier identification of neuropathy, improve adherence to therapeutic interventions, and optimize both neurological and CV outcomes [53,54,55,56].

Moreover, dietary interventions represent a key yet underexplored component in the management of patients with T2D, obesity, and DPN. Nutritional strategies targeting lipid metabolism, such as reducing saturated fatty acids and optimizing overall metabolic control, may influence both systemic inflammation and neuronal function. Educational interventions have been shown to improve cardiometabolic outcomes and patient adherence, highlighting the importance of structured dietary counseling. Recent evidence supports the role of dietary education within integrated care models, demonstrating improvements in long-term outcomes and risk factor control [57,58]. Future research should investigate the impact of targeted nutritional interventions on lipidomic profiles and the progression of neuropathy.

Importantly, future interventional trials should evaluate whether targeted modification of lipid metabolism can alter neuropathy progression or improve neurological outcomes. Potential strategies may include TG-lowering therapies, dietary interventions targeting saturated fatty acid intake, weight reduction programs, insulin-sensitizing therapies, and agents modulating mitochondrial function or lipid signaling pathways. Randomized controlled trials with long-term follow-up and standardized neuropathy outcomes are particularly needed.

Further research should also assess the feasibility and clinical utility of incorporating lipidomic biomarkers into routine risk stratification models for patients with obesity and T2D. Before translation into clinical practice, issues related to analytical standardization, reproducibility, cost-effectiveness, and accessibility of lipidomic technologies must be addressed in large and ethnically diverse populations.

### 4.8. Strengths and Limitations

The strengths of this review include a systematic methodological approach based on PRISMA guidelines and independent risk-of-bias assessment, integration of preclinical and clinical data with emerging lipidomic data, and integration of small mechanistic studies with large epidemiologic datasets. To our knowledge, this is the first systematic review specifically addressing lipid metabolism disturbances and advanced lipidomic alterations in the context of DPN in patients with both obesity and T2D. While previous studies have explored individual metabolic components, no prior synthesis has integrated conventional lipid parameters with emerging lipidomic data in this specific population.

This study has several limitations that should be considered when interpreting the findings. The fact that only 8 studies, from an initial pool of 452, met the inclusion criteria highlights a significant gap in the literature rather than a limitation of the search strategy and limits the robustness of the conclusions. The search strategy may have limited the retrieval of studies using broader neuropathy or lipidomic terminology, potentially contributing to the small number of eligible studies, along with the restriction to English-language publications that may have introduced selection bias. However, the narrower approach was intended to prioritize specificity and clinical relevance.

The generalizability of the results is limited by the heterogeneity of the included studies in terms of design, population characteristics, and methods used to assess lipid parameters and DPN, from clinical diagnosis to nerve conduction studies and intraepidermal nerve fiber density. Most studies were cross-sectional, precluding causal inference and limiting the ability to assess temporal relationships.

Advanced lipidomic analyses were often conducted in relatively small and highly selected cohorts, which may not be representative of the broader population of patients with T2D and obesity. In contrast, large epidemiological studies lacked detailed molecular profiling, limiting mechanistic interpretation.

Also, lipid abnormalities may contribute to endothelial dysfunction and impaired microcirculation, further exacerbating nerve injury—the role of microvascular dysfunction and its interaction with metabolic factors. At the same time, DPN is increasingly recognized as a multifactorial condition involving metabolic, vascular, and inflammatory mechanisms. Yet, the included studies rarely fully integrate these pathways, which represents an important gap in the literature.

Overall, the current evidence supports a multifactorial and metabolically interconnected model of DPN in which lipid abnormalities may interact with hyperglycemia, inflammation, mitochondrial dysfunction, and microvascular injury, although the available evidence remains insufficient to establish direct causality.

Importantly, most of the included studies were observational and predominantly cross-sectional, limiting the ability to establish temporal or causal relationships between lipid abnormalities and DPN progression. In addition, mechanistic lipidomic studies were generally exploratory and involved relatively small cohorts, increasing the risk of overinterpretation and limiting external validity. Variability in neuropathy phenotyping, lipidomic analytical platforms, and statistical adjustment strategies further reduced comparability across studies. Therefore, the current findings should be interpreted primarily as associative and hypothesis-generating rather than definitive evidence of causality. Due to these constraints, a meta-analysis was not feasible, and a narrative synthesis was considered the most appropriate approach. This decision reflects the heterogeneity of the data rather than a methodological limitation of the review itself.

## 5. Conclusions

Overall, the available evidence suggests that altered lipid metabolism, particularly elevated TG levels and lipidomic dysregulation, is associated with DPN in patients with obesity and T2D. However, the current literature remains largely observational and heterogeneous, limiting the ability to establish direct causal relationships between specific lipid abnormalities and neuropathy development or progression.

While TG > 204 mg/dL may be a potentially useful marker of DPN risk, its role in routine clinical practice requires further validation. Similarly, advanced lipidomic profiling offers promising research perspectives but is not yet applicable in standard clinical settings. Emerging advances in lipidomics and systems biology may substantially improve understanding of the complex metabolic pathways involved in DPN. As analytical technologies become more standardized and accessible, lipidomic profiling may eventually contribute to earlier risk stratification if validated in large prospective studies on patients with obesity and T2D. In particular, the integration of lipidomics with clinical, genetic, and inflammatory data may facilitate the identification of mechanistically relevant biomarkers and novel therapeutic targets.

Future research should move beyond cross-sectional associations and prioritize prospective longitudinal studies capable of clarifying temporal and potentially causal relationships between lipid abnormalities and neuropathy progression. Randomized controlled trials evaluating dietary interventions, TG-lowering therapies, insulin-sensitizing agents, and strategies targeting mitochondrial dysfunction or lipid signaling pathways are especially needed. Furthermore, harmonization of lipidomic methodologies, standardized neuropathy phenotyping, and the inclusion of ethnically and clinically diverse populations will be essential to improve reproducibility and translational applicability.

## Figures and Tables

**Table 1:** Newcastle–Ottawa Scale analysis of the included studies [16,21,22,23,24,25,26,27].

Author (Reference)	Selection				Comparability	Outcome			Total Score	Quality
	Representativeness of the Exposed Cohort	Selection of the Non-Exposed Cohort	Ascertainment of Exposure	Demonstration that Outcome of Interest Was Not Present at Start of Study	Comparability of Cohorts Based on the Design or Analysis	Assessment of Outcome	Was Follow-Up Long Enough for Outcomes to Occur	Adequacy of Follow-Up of Cohorts		
Wang et al. [21], 2025	-	+	+	+	+	+	+	-	6	Good
Kristensen et al. [22], 2024	+	-	+	+	+	+	+	+	7	Very good
Lin et al. [23], 2023	+	-	+	+	+	+	+	+	7	Very good
Rumora et al. [16], 2021	+	+	+	+	-	+	+	+	7	Very good
Afshinnia et al. [24], 2022	+	-	+	+	+	+	+	+	7	Very good
Callaghan et al. [25], 2016	+	-	+	+	-	+	+	+	6	Good
Callaghan et al. [26], 2016	+	-	+	+	-	+	+	+	6	Good
Daniele et al. [27], 2014	+	-	+	+	+	+	+	+	7	Very good

“+” indicates that the article meets the criteria mentioned above; “-” indicates that the article does not meet the above-mentioned criteria.

**Table 2:** Included studies’ characteristics and parameters of interest for adult patients with obesity and DPN [24,25,26].

First Author, Publication Year	Location	Sample	Groups	Clinical Outcomes		Statistical Power
Callaghan et al. [25], 2016	Michigan	155	102 obese group, 53 lean group	Association of neuropathy and TG	unit of 50 mg/dL	Unadjusted OR 1.06 (0.78, 1.45)
				Association of neuropathy and HDL-C	unit of 10 mg/dL	Unadjusted OR 0.97 (0.66, 1.43)
				Association of IENFD and TG	unit of 50 mg/dL	95% CI −0.23 (−0.56, 0.10)
				Association of IENFD and HDL-C	unit of 10 mg/dL	95% CI −0.56 (−1.03, −0.10)
Callaghan et al. [26], 2016	NR	2382	MetS and Neuropathy	Association of Neuropathy and TG	unit of 50 mg/dL	OR (95% CI) 1.01 (0.93, 1.10), *p* < 0.05.
				Association of Neuropathy and HDL-C	unit of 10 mg/dL	OR (95% CI) 0.91 (0.81, 1.01), *p* < 0.05.
				Association of incident Neuropathy and TG	unit of 50 mg/dL	OR 0.99 [95% CI 0.84–1.16]
				Association of Incident Neuropathy and HDL-C	unit of 10 mg/dL	OR 0.94 [95% CI 0.81–1.09]
Daniele et al. [27], 2014	Texas	22	11 obese group, 11 T2D group	fasting plasma FFA before and after 2 weeks acipimox	0.22 ± 0.04 vs. 0.043 ± 0.003 mmol/L	*p* not significant

TG—triglycerides; HDL-C—high-density lipoprotein cholesterol; IENFD—intraepidermal nerve fiber density; OR—odds ratio; CI—confidence interval; FFA—free fatty acids; T2D—type 2 diabetes.

**Table 3:** Included studies’ characteristics and parameters of interest for adult patients with T2D and DPN [16,21,22,23,27].

First Author, Publication Year	Location	Sample	Groups	Clinical Outcomes			Statistical Power
Wang et al. [21], 2025	Europe	7174	DPN present vs. PDN absent	An increase in phosphatidylcholine (16:0_20:2) decreases DPN risk		OR = 0.82, 95%CI: 0.73–0.91; FDR = 0.033	*p* < 0.001
				An increase in phosphatidylcholine (16:1_18:1) decreases DPN risk		OR = 0.77, 95%CI: 0.67–0.88; FDR = 0.019	*p* < 0.001
Kristensen et al. [22], 2024	NR	61,853	T2D and DPN	adjusted PRs (95% CI) for TG level > 204 mg/dL and DPN		1.40 (1.21–1.62)	NR
Lin et al. [23], 2023	China	998,379	T2D and DPN	Unadjusted PAF (%)	HbA1c ≥ 7%	14.2	NR
					BP ≥ 130/80 mmHg	11.7	NR
					LDL-C ≥ 1.8 mmol/L	5.9	NR
					BMI ≥ 24 kg/m^2^	5.8	NR
				Adjusted (by age, sex, and duration of DM) PAF (%)	HbA1c ≥ 7%	9.0	NR
					BP ≥ 130/80 mmHg	6.8	NR
					LDL-C ≥ 1.8 mmol/L	4.3	NR
					BMI ≥ 24 kg/m^2^	4.8	NR
Afshinnia et al. [24], 2022	Gila River Indian Community	69	T2D—27 with neuropathy and 42 without neuropathy	Total cholesterol (mg/dL)		162 ± 41 versus 165 ± 37	*p* = 0.788
				Triglyceride (mg/dL)		184 ± 160 versus 195 ± 240	*p* = 0.839
Rumora et al. [16], 2021	Denmark	106	Control 9 patients T2D without DPN 49 patients T2D with DPN 48 patients	Total cholesterol (mmol/L)		4.26 ± 0.96 versus 4.47 ± 0.83 versus 6.06 ± 1.93	*p* < 0.01 *p*= 0.5817
				TG (mmol/L)		1.93 ± 1.18 versus 1.69 ± 0.74 versus 1.12 ± 0.44	*p* = 0.2260 *p* = 0.4312
				Significant reduction		ximenoylcarnitine (26:1),	*p* < 0.05
						lignoceroylcarnitine (24:0)	*p* < 0.05
						glycosyl-N-(2-hydroxynervonoyl)-sphingosine (d18:1_24:1(2OH))]	*p* < 0.05
Daniele et al. [27], 2014	Texas	22	11 obese group, 11 T2D group	fasting plasma FFA before and after 2 weeks acipimox		0.25 ± 0.05 vs. 0.057 ± 0.008 mmol/L with	*p* < 0.05

T2D—type 2 diabetes; DPN—diabetic peripheral neuropathy; FDR—false discovery rate; PR—prevalence ratio; CI—confidence interval; TG—triglycerides; PAF—population attributable factor; BP—blood pressure; LDL-C—low-density lipoprotein cholesterol; BMI—body mass index; FFA—free fatty acids; NR—not reported.

**Table 4:** Proposed framework linking lipid dysregulation and lipidomic alterations with potential mechanisms involved in DPN.

Lipid metabolism disturbances			
↑ Triglycerides ↑ Free fatty acids Dyslipidemia (↓ HDL-C, ↑ LDL-C)	Lipidomic alterations: Acylcarnitines Sphingolipids Phospholipids		
Advanced lipidomic alterations			
**Mitochondrial dysfunction** Impaired β-oxidation ↓ ATP production ↑ Reactive oxygen species ↓Neuronal energy deficit	**Neuroinflammation and oxidative stress** Cytokine activation Lipotoxicity Oxidative damage ↓Axonal injury	**Neurovascular impairment** Endothelial dysfunction ↓ Microcirculation Ischemia/hypoxia ↓ Reduced nerve perfusion	
Peripheral nerve damage			
Axonal degeneration	Demyelination	Small fiber loss	Neuropathic pain
Metabolic and systemic modulators			
Hyperglycemia	Insulin resistance	Chronic inflammation	Metabolic syndrome

↑—increase; ↓—decrease.

## Data Availability

All data are within the article.

## Supplementary Materials

The following supporting information can be downloaded at: https://www.mdpi.com/article/10.3390/jcm15103976/s1, Table S1: PRISMA 2020 checklist.

## Abbreviations

The following abbreviations are used in this manuscript:

CV	Cardiovascular
DM	Diabetes mellitus
DPN	Diabetic peripheral neuropathy
FFAs	Free fatty acids
LPC	Lysophosphatidylcholine
LPE	Lysophosphatidylethanolamine
T2D	Type 2 diabetes
TGs	Triglycerides
